# Comparative host protein interactions with HTLV-1 p30 and HTLV-2 p28: insights into difference in pathobiology of human retroviruses

**DOI:** 10.1186/1742-4690-9-64

**Published:** 2012-08-09

**Authors:** Rami Doueiri, Rajaneesh Anupam, Mamuka Kvaratskhelia, Kari B Green, Michael D Lairmore, Patrick L Green

**Affiliations:** 1 Center for Retrovirus Research, The Ohio State University, Columbus, OH, 43210, USA; 2 Department of Veterinary Biosciences, The Ohio State University, Columbus, OH, 43210, USA; 3 Department of Molecular Virology, Immunology, and Medical Genetics, The Ohio State, University, Columbus, OH, 43210, USA; 4 Comprehensive Cancer Center and Solove Research Institute, The Ohio State University, Columbus, OH, 43210, USA; 5 College of Pharmacy, The Ohio State University, Columbus, OH, 43210, USA; 6 Mass Spectrometry and Proteomics Facility, The Ohio State University, Columbus, OH, 43210, USA; 7 Department of Pathology, Microbiology, and Immunology, University of California, Davis, CA, 95616, USA

## Abstract

**Background:**

Human T lymphotropic virus type-1 (HTLV-1) and type 2 (HTLV-2) are closely related human retroviruses, but have unique disease associations. HTLV-1 is the causative agent of an aggressive T-cell leukemia known as adult T-cell leukemia (ATL), HTLV-1 associated myelopathy/tropical spastic paraparesis (HAM/TSP), and other inflammatory diseases. HTLV-2 infection has not been clearly associated with any disease condition. Although both viruses can transform T cells *in vitro*, the HTLV-1 provirus is mainly detected in CD4+ T cells whereas HTLV-2 is mainly detected in CD8+ T cells of infected individuals. HTLV-1 and HTLV-2 encode accessory proteins p30 and p28, respectively, which share partial amino acid homology and are required for viral persistence *in vivo*. The goal of this study was to identify host proteins interacting with p30 and p28 in order to understand their role in pathogenesis.

**Results:**

Affinity-tag purification coupled with mass spectrometric (MS) analyses revealed 42 and 22 potential interacting cellular partners of p30 and p28, respectively. Of these, only three cellular proteins, protein arginine methyltransferase 5 (PRMT5), hnRNP K and 60 S ribosomal protein L8 were detected in both p30 and p28 fractions. To validate the proteomic results, four interacting proteins were selected for further analyses using immunoblot assays. In full agreement with the MS analysis two cellular proteins REGγ and NEAF-interacting protein 30 (NIP30) selectively interacted with p30 and not with p28; heterogeneous nuclear ribonucleoprotein H1 (hnRNP H1) bound to p28 and not to p30; and PRMT5 interacted with both p30 and p28. Further studies demonstrated that reduced levels of PRMT5 resulted in decreased HTLV-2 viral gene expression whereas the viral gene expression of HTLV-1 was unchanged.

**Conclusion:**

The comparisons of p30 and p28 host protein interaction proteome showed striking differences with some degree of overlap. PRMT5, one of the host proteins that interacted with both p30 and p28 differentially affected HTLV-1 and HTLV-2 viral gene expression suggesting that PRMT5 is involved at different stages of HTLV-1 and HTLV-2 biology. These findings suggest that distinct host protein interaction profiles of p30 and p28 could, in part, be responsible for differences in HTLV-1 and HTLV-2 pathobiology. This study provides new avenues of investigation into mechanisms of viral infection, tropism and persistence.

## Background

Human T lymphotropic virus type 1 (HTLV-1) and type 2 (HTLV-2) are complex deltaretroviruses that are closely related with approximately 70% nucleotide sequence similarity [1]. HTLV-1 was the first retrovirus linked to human malignancy [2,3]. HTLV-1 can infect T cells, B cells, fibroblasts and macrophages; however, the provirus is predominantly detected in CD4+ T cells [4-6]. HTLV-1 infection causes adult T-cell leukemia (ATL) in approximately 1–5% of infected individuals [7]. In addition, HTLV-1 infection has also been associated with a neurodegenerative disease, HTLV-1 associated myelopathy/tropical spastic paraparesis (HAM/TSP) and other immune-mediated inflammatory diseases [8]. Despite being a closely related retrovirus, HTLV-2 unlike HTLV-1, has no clear disease association with the exception of a few cases of HAM/TSP-like neurodegenerative disease [9]. HTLV-2 shows selective tropism towards CD8+ T cells, both *in vitro* and *in vivo*[10-13]. Furthermore, although both viruses are capable of transforming T cells *in vitro*, it is intriguing that they exhibit sharply distinct target cell and transformation tropisms, and only HTLV-1 is associated with malignancy and chronic inflammatory conditions [14,15].

The genome organization of the HTLV-1 and HTLV-2 provirus is very similar with 5’ and 3’ LTRs. Both viruses encode regulatory and accessory proteins apart from the typical structural and enzymatic proteins Gag, Pol and Env. Two key regulatory gene products are Tax-1/Tax-2 and Rex-1/Rex-2 corresponding to HTLV-1/HTLV-2, respectively.

The Tax proteins encoded by both viruses are considered the primary oncoproteins [16,17] required for T cell transformation. However, Tax is not sufficient for the malignant process; additional proteins encoded by the virus are suggested to play a role as well [18]. For instance, HTLV-1 and HTLV-2 encode HZB and APH-2 proteins from the antisense strand of the proviral genome respectively [19,20], and growing evidence indicates a role for HBZ in the transformation process [21].

HTLV-1 p30 and HTLV-2 p28 are accessory proteins involved in the regulation of viral replication and persistence and could therefore affect the pathogenic outcome [22-27]. Both proteins are encoded by the corresponding viruses from a doubly spliced mRNA from the ORFII of the pX region and are dispensable for *in vitro* viral infection and T cell transformation. Interestingly, both p30 and p28 are required for the establishment of viral persistence in the rabbit model, underlining the importance of these proteins *in vivo*[25,27,28]. In addition, p30 has been shown to be required for infection of dendritic cells and non-human primates [26]. Both p30 and p28 are nuclear/nucleolar localizing proteins [29,30]. The nucleolar retention of p30 is linked to RNA transcription and binding to the 60 S ribosomal subunit [31]. Both p30 and p28 are post-transcriptional negative regulators of viral gene transcription that act by retaining *tax/rex* mRNA in the nucleus [22,30]. However, evidence indicates that p30 also regulates viral gene expression at a transcriptional level by competing with Tax for binding to CBP/p300 [23]. In addition, p30 and Rex may be a part of the nuclear retention mechanism by forming a ribonucleoprotein complex with *tax/rex* mRNA [32].

Microarray studies and genome-wide screens have shown that p30 differentially modulates cellular gene expression [33,34]. Expression of p30 activates the G2/M cell cycle checkpoint to promote cell survival, and delays entry into S-phase [35,36]. Under genotoxic stress, p30 promotes cell survival by binding and modulating levels of ATM possibly through binding to REGγ [37]. The ability of p30 to bind to the Myc-Tip60 complex and to also promote non-homologous end joining DNA repair support its role in cellular transformation [38,39].

Comparative studies of host protein interactions with HTLV-1 and HTLV-2 proteins have been largely focused on Tax-1 and Tax-2 [18,40-42]. Studies comparing host protein interactions of HTLV-1 and HTLV-2 accessory proteins have not been performed. Herein, we compared the cellular interacting protein profiles of p30 and p28 to better understand their roles in viral infection, persistence and cellular transformation. To this end, we used affinity tag purification of p30 and p28 coupled to mass spectrometry to identify potential interacting cellular proteins. We have confirmed the interaction of p30 with REGγ and identified a new p30 binding partner, NEAF-interacting protein 30 (NIP30). These cellular proteins copurified with p30 and not p28. In contrast, heterogeneous nuclear ribonucleoprotein H1 (hnRNP H1) interacted with p28 and not p30. Our data also reveal that arginine methyl transferase 5 (PRMT5) can interact with both p30 and p28. Knockdown studies of PRMT5 have indicated that this protein is important for effective gene expression of HTLV-2 and not HTLV-1. Our data provide new insights into the comparable host cell protein interactions used by these closely related human retroviruses.

## Results

### Host protein interaction profiles of HTLV-1 p30 and HTLV-2 p28

In order to sample the p30 and p28 cellular proteome, we employed S-tag affinity purification [43] of ectopically expressed HTLV-1 p30 and HTLV-2 p28 in HEK 293T cells. An amino terminal S-tag and a HA and AU1 tag on the carboxy terminus were added to a CMV driven pTriEx4-Neo plasmid. S-tag affinity pull-down was performed on the lysates of cells, transfected with either empty vector (mock control) or p30 or p28, using S-beads. We analyzed p30- and p28-associated proteins using shotgun proteomics. The proteins that were unique to p30 and p28 purification fractions after subtracting the mock control proteins were considered as their potential interacting partners. The data were further refined by eliminating contaminants and highly abundant proteins, such as keratin, that were detected in controls. Duplicate experiments resulted in the identification of 42 and 22 potential interacting partners of p30 and p28, respectively (Tables 1 and 2). Of these, only three cellular proteins, PRMT5, hnRNP K and 60 S ribosomal protein L8 were detected in both p30 and p28 fractions. In order to validate the results of mass spectrometry-based proteomic experiments, we selected the following four cellular proteins for immunoblotting assays: two proteins REGγ and NEFA-interacting nuclear protein NIP30 (NIP30), which were exclusively found in p30 fractions (Table 1); heterogeneous nuclear ribonucleoprotein H1 (hnRNP H1) that purified with p28 and not with p30; and protein arginine methylate transferase 5 (PRMT5), which was found in both p30 and p28 fractions (Tables 1 and 2). An additional negative control of amino terminal S-tag GFP (S-GFP) expressed from the same expression vector was also tested in the immunoblotting assays.

**Table 1:** HTLV-1 p30-interacting host proteins

**Name of the protein**	**Molecular function**	**Unweighted spectrum count**	**% coverage**
Proteasome activator complex subunit	Cell cycle and protein degradation	21	54
Heat shock protein 90 beta	Protein folding	9	14
Heat shock protein 90 alpha	Protein folding	7	8.2
Methylosome subunit pICln	Methylosome component	5	37
Reticulocalbin-2	Calcium binding	5	20
Succinate dehydrogenase [ubiquinone] flavoprotein subunit	Electron transport	5	9.2
Elongation factor Tu	Translation	5	17
Clathrin heavy chain 1	Vesicular transport	5	5.4
Protein arginine N-methyltransferase 5	Methyltransferase activity	4	16
Cofilin-1	Cytoskeleton organization	4	27
Malate dehydrogenase	Citric acid cycle	4	14
Protein phosphatase 1 G	Phosphatase activity	4	11
Importin-5	Nuclear import	3	3
Ubiquitin	Post translation modification	3	17.4
14–3–3 protein zeta/delta	Signaling pathways	3	17
Complement component 1 Q subcomponent-binding protein	Immune response	3	25
L-lactate dehydrogenase A chain	Glycolysis	3	13
Methylosome protein 50	Methylosome component	2	7.6
NEFA-interacting nuclear protein NIP30	Unknown	2	9.8
Fatty acid synthase	Fatty acid metabolism	2	0.68
Transgelin-2	Predicted muscle development	2	15
Alpha-1-antiproteinase	Serine protease inhibitor	2	7.7
DNAJ homolog subfamily A member 1	Chaperon activity	2	13
Ubiquitin-conjugating enzyme E2 L3	Ubiquitination activity	2	24
F-box only protein 22	Ubiquitination activity	2	6.2
Phosphoglycerate mutase 1	Glycolysis	2	13
Peptidyl-prolyl cis-trans isomerase	Protein folding	2	11
60 S ribosomal protein L3	Component of ribosome	2	8.7
60 S ribosomal protein L8	Component of ribosome	2	16
Hydroxyacyl-coenzyme A dehydrogenase	Fatty acid metabolism	1	9.6
Nucleosome assembly protein 1-like 1	Predicted nucleosome assembly	1	7.4
Arginyl-tRNA synthetase	Arginine tRNA ligation	1	2
Glucose-6-phosphate isomerase	Glycolysis	1	2.7
Apoptosis-inducing factor 1	Apoptosis	1	1.8
Mesencephalic astrocyte-derived neurotrophic factor	Neuronal growth factor	1	6.1
Inorganic pyrophosphatase 2	Inorganic phosphatase activity	1	4.8
Tetratricopeptide repeat protein 4	Predicted binding activity	1	2.6
Myosin-9	Cytoskeletal organization	1	0.82
Heterogeneous nuclear ribonucleoprotein K	mRNA processing	1	2.6
14–3–3 protein epsilon	Signaling pathways	1	11
Profilin-1	Cytoskeletal organization	1	10
60 S ribosomal protein L30	Component of ribosome	1	10

List of host proteins identified by shotgun proteomics that co-purified with p30 after eliminating non-specific and contaminating proteins. The molecular processes in which these proteins participate are indicated. The numbers of unweighted spectrum counts and the percentage of coverage of each protein are shown.

**Table 2:** HTLV-2 p28-interacting host proteins

**Name of the protein**	**Molecular function**	**Unweighted spectrum count**	**% coverage**
Heterogeneous nuclear ribonucleoprotein H1	mRNA processing	8	4.3
Heterogeneous nuclear ribonucleoprotein K	mRNA processing	6	5.2
Serine/threonine-protein phosphatase 6 catalytic subunit	Protein phosphatase	5	8.9
Serine/threonine-protein phosphatase 6 regulatory subunit 3	Phosphatase regulator	3	5.1
Protein arginine methyl transferase 5	Arginine methylation	3	6.4
Poly (A) binding protein 1 or 4	Poly (A) RNA binding	3	12
Inorganic pyrophosphatase	Inorganic diphosphatase activity	2	9
Peroxiredoxin-2	Antioxidant	2	15
60 S ribosomal protein L29	Component of ribosome	2	9.3
60 S ribosomal protein L8	Component of ribosome	2	5.2
Nucleolin	RNA,DNA and nucleotide binding	1	1.4
Glutathione S-transferase P	Glutathione transferase activity	1	13
Serine/threonine-protein phosphatase 2A regulatory subunit B	Phosphatase regulator	1	3.3
Nascent polypeptide-associated complex subunit alpha-2	Protein transport	1	7
Cyclic nucleotide gated channel 3	Predicted ion channel activity	1	1.7
Obg-like ATPase 1	Predicted ATP hydrolysis	1	3.8
Elongation factor Tu (Mitochondrial)	Translation elongation	1	2.9
Triosephosphate isomerase	Phosphate isomerase	1	9.6
Heparan sulfate proteoglycan 2	Predicted extracellular matrix binding	1	0.34
Heterogeneous nuclear ribonucleoprotein F	RNA processing	1	4.1
40 S ribosomal protein S8	Component of ribosome	1	5.4
Chaperonin 10	Protein folding	1	11

List of host proteins identified by shotgun proteomics that co-purified with p28 after eliminating non-specific and contaminating proteins. The molecular processes in which these proteins are involved are mentioned next to the proteins. The number of unweighted spectrum counts and percentage coverage of each protein are indicated.

### REGγ and NIP30 interact with p30 and not with p28

**Figure 1:**
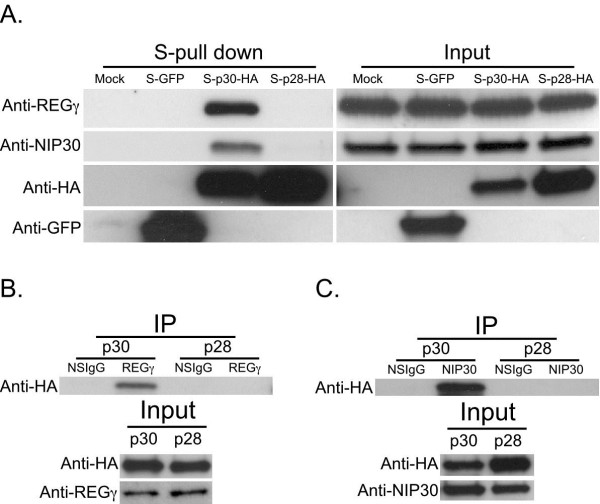
**Validation of p30 interaction with REGγ and NIP30.A**) S-tag affinity purification with Mock, S-GFP, S-p30-HA and S-p28-HA transfected 293T cell lysates. The purified product was analyzed by immunoblotting using indicated antibodies. The expression of individual protein and S-tag purification was confirmed using the indicated antibodies. **B**) 293T cells were transfected with S-p30-HA and S-p28-HA and immunoprecipitated with non-specific IgG (NSIgG) or anti-REGγ antibody and probed with anti-HA antibody. Immunoblotting of input using anti-REGγ and anti-HA antibodies confirmed the expression of the interacting proteins. **C**) 293T cells lysates expressing S-p30-HA and S-p28-HA were immunoprecipitated with non-specific IgG (NSIgG) or anti-NIP30 antibody and probed with anti-HA antibody. The expression of p30, p28 and NIP30 was confirmed with indicated antibodies.

We previously identified the interaction of p30 and REGγ using proteomic and molecular biology techniques [37]. However, the interaction of p28 and REGγ was not investigated previously. The proteomic data reported here indicate that REGγ selectively interacts with p30 and not with p28 (Tables 1 and 2). Similarly, we detected NIP30 in p30 (Table 1) and not in p28 (Table 2) fractions. To further evaluate these observations, 293T cells were transfected with mock, S-GFP, S-p30-HA and S-p28-HA. The cell lysates were subjected to S-tag affinity purification and immunoblotted with anti-REGγ and NIP30 antibodies. As shown in the Figure 1A, co-purification of REGγ and NIP30 with p30 and not p28 indicates the specific interaction of these proteins with p30. The expression and S-tag enrichment of p28 and p30 was tested by immunoblotting with anti-HA antibodies, whereas the expression and S-tag enrichment of GFP was evaluated with anti-GFP antibodies (Figure 1A).

The interaction of p30 with REGγ was further confirmed by immunoprecipitating REGγ from lysates of cells transfected with S-p30-HA and S-p28-HA and immunoblotted with anti-HA antibody. REGγ was able to co-immunoprecipitate p30 but not p28 (Figure 1B). These results are fully consistent with our proteomic data (Tables 1 and 2). Similarly, we confirmed the interaction of NIP30 with p30 by immunoprecipitating NIP30 from S-p30-HA and S-p28-HA transfected cell lysates. The results shown in Figure 1C indicate that NIP30 can selectively co-immunoprecipitate p30 but not p28. Immunoblotting of the cell lysate with anti-REGγ, anti-NIP30 and anti-HA antibodies confirmed the expression of endogenous REGγ and NIP30 along with transfected p30 and p28 (Figure 1B, C). To evaluate the functional significance of these interactions we knocked down REGγ and NIP30 using siRNA, then transfected HTLV-1 molecular proviral clone plasmid, and measured p24 production. We observed that reduced levels of REGγ and NIP30 had no effect on p24 levels ( Additional file 1: Figure S1).

### p28 exclusively interacts with hnRNP H1

**Figure 2:**
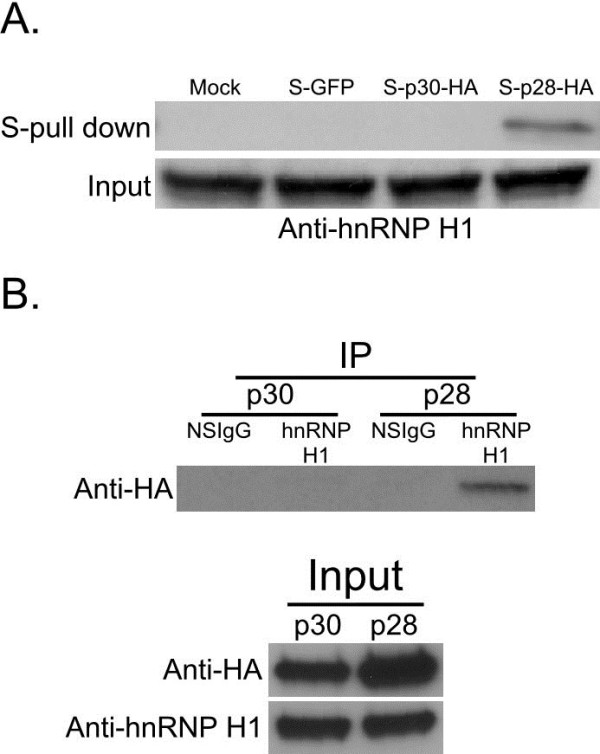
**Validation of p28 interaction with hnRNP H1.A**) Ectopically expressed Mock, S-GFP, S-p30-HA, S-p28-HA in 293T cells were S-tag affinity purified and probed with anti-hnRNP H1 antibodies. Endogenous expression of hnRNP H1 was confirmed by immunoblotting with anti-hnRNP H1 antibody. **B**) The immunoprecipitation assay was performed on cell lysates transfected with S-p30-HA and S-p28-HA. Non-specific IgG (NSIgG) or anti-hnRNP H1 antibodies were used for immunoprecipitation and subsequently probed with anti-HA antibody. Immunoblotting using anti-HA and anti-hnRNP H1 was used to validate the expression of all proteins.

To confirm the interaction of p28 with hnRNP H1, lysates from cells with transient expression of mock, S-GFP, S-p30-HA and S-28-HA were S-tag affinity purified and immunoblotted with anti-hnRNP H1 antibody. The presence of hnRNP H1 only in the p28 purification and not in GFP or p30 purifications (Figure 2A) indicates that hnRNP H1 specifically binds to p28. Immunoblotting with anti-GFP and anti-HA to evaluate the expression and affinity purification of transfected GFP, p30 and p28 is shown in Figure 1A. In order to further confirm the interaction between p28 and hnRNP H1, lysates from cells transfected with S-p30-HA and S-p28-HA were immunoprecipitated with anti-hnRNP H1 antibodies and probed with anti-HA antibodies. Selective co-immunoprecipitation of p28 and not p30 by hnRNP H1 (Figure 2B) indicates that only p28 interacts with hnRNP H1.

### p30 and p28 interact with PRMT5

**Figure 3:**
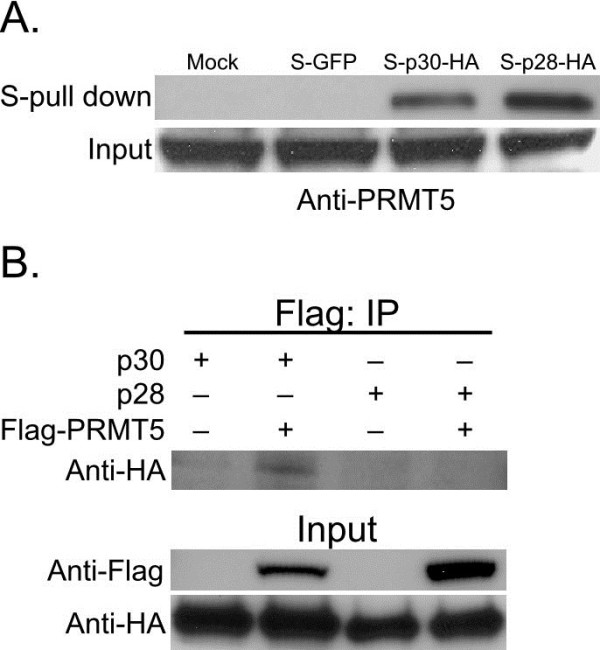
**Validation of p30 and p28 interaction with PRMT5.** Mock, S-GFP, S-p30-HA and S-p28-HA expressed in 293T cells were purified by S-tag affinity purification and immunoblotting with anti-PRMT5 antibody. The expression of PRMT5 was confirmed by immunoblotting the cell lysates with anti-PRMT5 antibody. **B**) 293T cells co- transfected with p30 or p28 or Flag PRMT5 as indicated in the figure. Immunoprecipitation was performed using anti-Flag antibodies and subsequently probed for p30 and p28 using anti-HA antibodies. The expression of Flag-PRMT5, p30 and p28 was validated by immunoblotting as shown in the figure.

The mass spectrometry-based proteomic data indicate that both p30 and p28 interact with PRMT5. It should be noted that these experiments were conducted using S-tag affinity purification of lysates of 293T cells transfected with mock, S-GFP, S-p30-HA or S-p28-HA. While it has been reported [44] that PRMT5 can non-specifically associate with Flag-beads, our data show that with the S-tag affinity pull-down approach PRMT5 was detected in p30 and p28 fractions but not with mock or GFP purification. In order to confirm this interaction, S-tag affinity purification was performed on cell lysates from empty, S-GFP, S-p30-HA or S-p28-HA transfected 293T cells and analyzed by Western blotting using PRMT5 antibody. The ability of both p30 and p28 to co-purify PRMT5 (Figure 3) indicates that PRMT5 interacts with both p30 and p28. Immunoblots (Figure 1A) probed with anti-GFP and anti-HA antibodies confirmed the expression and S-tag purification of GFP, p30 and p28. To further validate the interaction of PRMT5 with p30 and p28, Flag tagged PRMT5 (Flag-PRMT5) was cotransfected with p30 or p28. Immunoprecipitation of Flag-PRMT5 using anti-Flag antibodies was able to co-immunoprecipitate p30 (Figure 3B). However, Flag-immunoprecipitation of Flag-PRMT5 was not able to co-immunoprecipitate p28 (Figure 3B). It is possible that the structural features of p28 allow the detection of PRMT5 binding only by S-tag affinity purification and not by immunoprecipitation. Looking at p30 proteomics data, it is also possible that interaction of p30 with PRMT5 binding proteins such as methylosome subunit pICln and methylosome 50 facilitate co-immunoprecipitation of p30 with PRMT5. The expression of Flag-PRMT5, p30 and p28 was confirmed using anti-Flag and anti-HA antibodies (Figure 3B).

### PRMT5 is required for HTLV-2 gene expression

**Figure 4:**
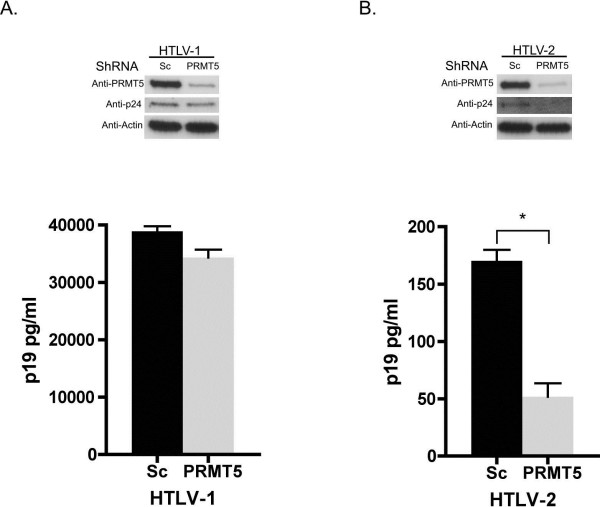
**PRMT5 knockdown and HTLV gene expression.A**) 293T cells expressing normal and knockdown levels of PRMT5 were transfected with HTLV-1 molecular clone. Immunoblotting was performed using anti-PRMT5 antibodies to confirm the knockdown and with anti-HTLV p24 antibodies to monitor p24 levels. The amount of p19 Gag production was analyzed by p19 ELISA. **B**) 293T cells transduced with scrambled shRNA or shRNA against PRMT5 were transfected with HTLV-2 molecular clone. Knockdown of PRMT5 was confirmed by immunoblotting with anti-PRMT5 antibodies. The intracellular p24 and extracellular p19 Gag production was analyzed by immunoblotting with anti-HTLV p24 antibodies and with p19 ELISA respectively. Student *t*-test resulted in a *p* value of 0.011 (_*_) indicating that decrease in p19 production with lower levels of PRMT5 is significant. Immunoblotting with anti-Actin antibodies was used to validate equal loading.

To investigate a potential role of PRMT5 in HTLV biology, we downregulated the protein levels in 293T cells using the shRNA approach. Cells were then transfected with either HTLV-1 or HTLV-2 molecular clone plasmids, and viral gene expression was measured by immunoblotting for intracellular p24 and by ELISA for p19 production. The levels of PRMT5 had little effect on HTLV-1 gene expression in terms of intracellular p24 and p19 production as shown in Figure 4A. However, lower levels of PRMT5 resulted in a significant reduction in intracellular HTLV-2 p24 (Figure 4B). Consistent with this result HTLV-2 p19 measured by ELISA was also reduced 3 fold in PRMT5 knockdown cells (Figure 4B). These results indicate that PRMT5 plays a role in HTLV-2 gene expression, but has no measurable effect on HTLV-1 gene expression.

## Discussion

HTLV-1 and -2 are closely related human retroviruses with HTLV-1 being pathogenic whereas a clear correlation between HTLV-2 and pathogenesis has not been established. HTLV-1 and HTLV-2 have different pathological outcomes that cannot be attributed to a single viral protein, but are likely due to the contribution of viral proteins and their interactions with the host cellular machinery. Both viruses encode accessory proteins that are required for viral persistence *in vivo*. We hypothesize that differences in viral infection, tropism and pathological outcome may result in part from how these accessory proteins interact with host proteins. Recently host-pathogen interactome study comparing HTLV-1 and HTLV-2 was reported to propose novel hypothesis to explain differential pathobiology of these closely related viruses [45]. However, in this study yeast-two hybrid screening did not result in any p30 or p28 interacting proteins. In our study we conjugated over expression and mass spectrometry to compare the host protein interaction proteome of HTLV-1 p30 and HTLV-2 p28 accessory proteins. There is approximately 77% homology between the last and first 49 amino acids of p30 and p28, respectively, whereas the remaining amino acid sequences of p30 and p28 differ markedly. Therefore, it is logical to propose that these two viral proteins may exhibit some similarities and differences in their interactions with cellular proteins.

We adapted S-tag purification of p30 and p28 and combined it with mass spectrometry to identify potential host protein interactions. The approach yielded the list of proteins (Table 1 and 2), with the number of host proteins interacting with p30 being higher than p28. These findings are consistent with the observation that p30 is a multifunctional protein regulating transcription and post transcriptional gene expression, cell cycle/cell survival and DNA damage [35-39,46]. In contrast, p28 has only been implicated in post transcriptional gene regulation [30]. However, both proteins are important for high proviral loads and persistence in the rabbit model.

**Figure 5:**
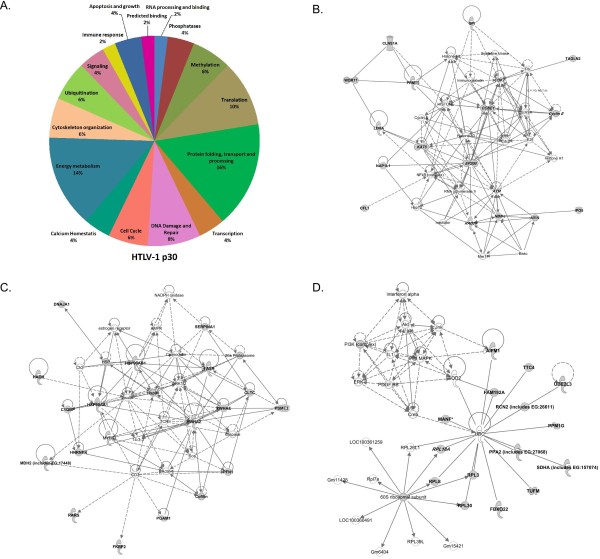
**Analysis of p30 interacting host proteins.A**) Functional distribution of HTLV-1 p30-interacting proteins summarized in the pie chart graph as percentages. **B**), **C**) and **D**) Ingenuity pathways analysis results in three network pathways with broad cellular functions with the involvement of p30-interacting proteins. The proteins that were identified in this study to interact with p30 are indicated in bold, and previously identified p30-interacting proteins are indicated in bold italics. The interaction between two proteins is indicated with a straight line; arrows indicate action upon in the direction of the arrow; and dashed lines indicate indirect interactions.

**Figure 6:**
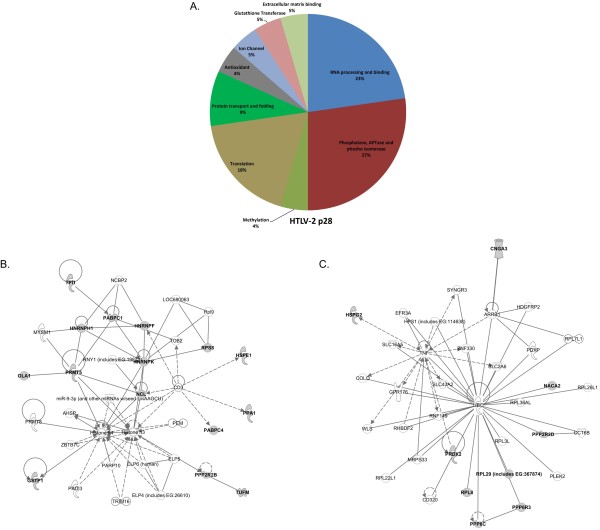
**Analysis of p28 interacting host proteins.A**) Functional distribution of HTLV-2 p28-interacting proteins summarized in the pie chart graph as percentages. **B**) and **C**) Ingenuity pathways analysis results in two network pathways with broad cellular functions with the involvement of p28-interacting proteins. The proteins that were identified in this study to interact with p28 are indicated in bold. The interaction between two proteins is indicated with a straight line; arrows indicate action upon in the direction of the arrow; and dashed lines indicate indirect interactions.

Detailed analysis of differences and similarities between p30 and p28 host protein-interacting profiles was performed in this study. For the analysis of host proteins that interact with p30, we combined the proteins identified in this study (Table 1) and previously identified proteins (Table 3). We then analyzed the molecular processes in which these interacting proteins are directly or indirectly involved. The graphical representation of molecular processes in which the host proteins interact with p30 (Figure 5A) and p28 (Figure 6A) suggests that p30 is involved in a wider variety of molecular processes than p28. The p30 pie graph (Figure 5A) indicates that 32% of p30 interacting proteins are involved in protein processes such as post translational modification (phosphorylation, ubiquitination and methylation), folding, and transport. Proteins involved in DNA damage repair and cell cycle constitute 14%, whereas proteins that are involved in energy metabolism contributed to 14% of the p30 interactome. Transcriptional proteins accounted for 4%, and only 2% of the proteins are involved in mRNA processing.

**Table 3:** Previously identified HTLV-1 p30 host interacting proteins

**Name of the protein**	**Molecular function**	**Reference**
Histone acetyltransferase p300	Transcription factor	[23,47]
60 S ribosomal protein L18a	Ribosomal component	[31]
Tat interacting protein 60 (TIP60)	Transcription and DNA damage repair	[38]
Ataxia telangiectasia mutated (ATM)	DNA damage repair	[37]
Nijmegen breakage syndrome protein 1(NBS1)	DNA damage repair	[39]
RAD 50	DNA damage repair	[39]
Cyclin E	Cell cycle	[36]
Cyclin dependent kinase 2 (CDK2)	Cell cycle	[36]

List of host proteins that were previously identified. The molecular processes in which these proteins are involved are indicated. The references to the studies that identified these interactions are also indicated.

The distribution of p28 interacting processes from Table 3 resulted in a lower number of molecular processes. The major molecular processes (23%) are RNA processing, while 27% of the proteins are involved in regulation of protein phosphorylation, folding and transportation. Translation contributes to about 10 and 18% of the p30 and p28 interactome, respectively. Binding of both p30 and p28 to phosphatases suggests that both proteins might be phosphorylated or modulate the phosphorylation status of their binding partners. The ability of p30 to interact with ubiquitin and with proteins having ubiquitin conjugation activity suggests that p30 might be ubiquitinated. p30 is involved in DNA damage repair mechanisms, and regulation of phosphorylation and ubiquitination play a critical role in DNA damage repair. A large number of p30-interacting proteins are involved in energy metabolism like electron transport, glycolysis and fatty acid synthesis, and further investigation should be carried out to understand the functional significance of these interactions.

Furthermore, we evaluated indirect involvement of p30 and p28 interacting host proteins in various cellular processes using Ingenuity Pathways Analysis (Ingenuity Systems, www.ingenuity.com). The analysis of all proteins that interact with p30 from Table 1 and with previously known p30-interacting proteins (Table 3) yielded three networks with high scores. The first network (Figure 5B) suggests significant involvement of p30-interacting proteins with DNA recombination, repair, cell cycle and cancer with pathways clustering around ATM, cyclin E, cyclin A and CDK2. It is consistent with various studies indicating the role of p30 in DNA damage repair, cell survival and cell cycle alteration. The second network (Figure 5C) revealed the influence of p30-interacting proteins on cancer, gastrointestinal disease and drug metabolism. The major convergence of the pathway was on ERK 1/2, Hsp 90 and 14–3–3 family proteins (YWHAZ). The last pathway (Figure 5D) is involved in neurological disease, developmental and hereditary disorders with central proteins being UBC, p38 MAPK and Akt, which are involved in various pathological conditions.

Similar analysis was performed using the list of p28-interacting proteins (Table 3) that resulted in two network pathways with high scores. The first pathway (Figure 6B) with histone proteins and hnRNP K at the center of the pathway suggested the involvement of p28-interacting proteins in RNA post transcriptional modification, amino acid metabolism and post translational modifications. The second pathway (Figure 6C) suggested the involvement of p28-interacting protein in neurological disease, skeletal muscle and development disorders. The central molecules of the second pathway were UBC and TNF, which are important for protein regulation and immune responses, respectively. The analysis suggests that p30 influences various pathways involved in processes such as cell cycle regulation, cell signaling and cancer biology. Interestingly, these pathways are also modulated by Tax and/or HBZ oncoproteins during cellular transformation. On the other hand, p28 has limited involvement in the kinds of cellular process that facilitate transformation.

We previously reported that p30 interacts with REGγ [37], which binds and activates the 20 S proteasome [48]. While REGγ targets a number of cellular proteins to proteasome degradation [49-52], we have shown that the levels of p30 correlate with the levels of REGγ indicating that p30 is not targeted to degradation via its interaction with REGγ [37]. We propose that the cell survival effect of p30 under naïve and genotoxic conditions is likely to be mediated through its interaction with REGγ to allow the viral infected cell to proliferate. Furthermore, our previous studies suggest that p30, ATM and REGγ are part of a multiprotein complex, and reduced levels of ATM under genotoxic stress might be due to REGγ binding to promote cell survival [37]. siRNA knockdown of REGγ had no effect on HTLV-1 viral gene expression ( Additional file 1: Figure S1A) suggesting that interaction of p30 with REGγ could play a role at other stages of the virus life cycle such as viral spread. In contrast with p30, p28 does not interact with REGγ. This difference between p30 and p28 could have possible long term effects during latency and clonal expansion of infected cells leading to the fully transformed or malignant state.

Another cellular protein NIP30, which selectively interacted with p30 and not with p28, is predicted to bind the DNA binding/EF hand/Leucine zipper protein (NEFA). NEFA was initially thought to bind DNA; however, it has been shown to be a Golgi localized calcium-binding protein [53,54]. The existence of NIP30 has been confirmed at the protein level and was initially identified by cDNA screens. It is localized to the nucleus, which is also the cellular compartment of p30 [55]. The biological role of NIP30 is not yet clear; however, based on its NEFA interaction it might play a role in calcium homeostasis. As calcium plays a crucial role in the immune response, the binding of p30 to NIP30 might influence the immune response to facilitate viral infection or replication [56]. Similarly to REGγ, NIP30 siRNA knockdowns had no effect on HTLV-1 viral gene expression ( Additional file 1: Figure S1B) suggesting that NIP30 might be involved in viral biology other than gene expression.

Although both p30 and p28 specifically bind *tax/rex* mRNA (potentially at exon 2/exon 3 spice junction) and retain it in the nucleus and thus regulate viral gene expression at the post transcriptional level, our findings in Figure 2 reveal that p28 and not p30 interacts with hnRNP H1, which is a part of spliceosome and involved in regulating splicing of mRNAs [57]. At the same time, the proteomics data show that both p30 and p28 bind to another host protein, hnRNP F, which is not only involved in mRNA processing but also has the ability to bind DNA [58,59]. Thus there is some degree of convergence and divergence in the mechanisms of action of p30 and p28. The processing of mRNA plays a critical role in viral infection and spread. The data suggest that p28 is more closely associated with mRNA processing compared to p30. However, both proteins interact and retain *tax/rex* mRNA in the nucleus as a part of the spliceosome to regulate viral gene expression. Further studies should provide insights into the mechanism of p30 and p28 function and avenues to develop novel therapeutic targets.

Both p30 and p28 interact with PRMT5, a type II PRMT enzyme that symmetrically dimethylates arginines [60]. The interaction of PRMT5 is of particular interest because it is up-regulated in B cell leukemia [61]. In the nucleus, PRMT5 is recruited to various transcription repressor complexes and also methylates histones H2A, H4R3 and H3R8 [62-65]. The methylation preference of PRMT5 is shifted to H4R3 upon binding to cooperator of PRMT5 (COPR5) [66]. A correlation between symmetrical methylation of H4R3 and H4R8 and transcription repression indicates the role of PRTM5 in transcription repression. PRMT5 also promotes cell survival via regulating p53 expression [67]. Upon DNA damage PRMT5 is known to methylate p53 through Strap co-factor to affect gene specificity of p53 [68]. In addition, PRMT5 functions in spliceosome formation and mRNA processing. PRMT5 is required for proper methylation of the mammalian cleavage factor 1 (mCF1), which is responsible for RNA 3’end processing [69]. PRMT5 is also required for Sm U snRNP methylation, which is important for *in vivo* for SMN complex formation to join proteins and RNA [70].

To investigate the possible role of PRMT5 interaction with p30 in transcriptional regulation, we performed PRMT5 knockdowns. HTLV-1 gene expression did not change significantly under low levels of PRMT5 (Figure 4A). The expression of p30 has been linked to increased cell survival and cell cycle deregulation. PRMT5 also has been linked to increased cell survival and to altering p53 function. Therefore, we posit that p30 and PRMT5 interaction might be relevant for cell survival to facilitate either viral spread or transformation. However, p28 interaction with PRMT5 might be relevant at transcriptional level by modulating the interaction of PRMT5 with transcription machinery. In addition p28 and PRMT5 interaction could also play a role at the post-transcriptional level in spliceosome formation, protein-mRNA recruitment or RNA processing. In both cases, PRMT5 would be affecting viral gene expression. This notion is consistent with our findings showing that downregulation of PRMT5 resulted in the reduced levels of HTLV-2 gene expression (Figure 4B). The interaction of p28 with PRMT5 might be regulating PRMT5 to modulate viral gene expression at either a transcriptional or post-transcriptional level. Furthermore, it is noteworthy that PRMT5 levels are up-regulated in B cell leukemia suggesting that PRMT5 might have a role to play in T cell transformation of HTLV-1 infected T cells. The study of the interaction of p30 and p28 with PRMT5 would provide a molecular model to understand the role of PRMT5 in cellular transformation.

## Conclusion

In summary, we identified 42 and 22 potential cellular proteins interacting with HTLV-1 p30 and HTLV-2 p28, respectively. Of these, only three cellular proteins overlapped indicating markedly different interacting profiles for these two viral proteins. PRMT5 interacted with both p30 and p28, but had a differential effect on the gene expression of HTLV-1 and HTLV-2 indicating that PRMT5 might have different roles in HTLV-1 and HTLV-2 viral biology. The differences in protein and cellular processes with which p30 and p28 engage could be one reason for the difference in viral infection outcomes. Elucidating the role of PRMT5 in HTLV-1 viral biology would provide insights into how HTLV-1 and HTLV-2 are utilizing a host protein differently to potentially result in different pathological outcomes. Further investigation to understand the functional relevance of these interactions is warranted to better understand the HTLV life cycle and its role in T-cell transformation in order to develop new therapeutic drug targets.

## Methods

### Cell culture and plasmid transfection

HEK 293T cells were cultured in DMEM containing 10% FBS, 2 mM L-glutamine, 100 mg/ml streptomycin, and 100 units/ml penicillin. Plasmids were transfected using SuperFect (Qiagen, Valencia, CA) according to the manufacturer’s instructions. Briefly 2x10^6^ cells were plated in 100 mm dishes and after 24 hr, cells were transfected with 10 μg of pTriEx4-Neo (Mock), 10 μg of pTriEX-Neo-S-GFP (S-GFP), 10 μg of pTriEx4-Neo-S-p30-HA (S-p30-HA), 10 μg of pTriEx4-Neo-S-p28-AU1 or 4 μg of pTriEx4-Neo-S-p28-HA(S-p28-HA) from Novagen, Madison, WI. For Flag-PRMT5 immunoprecipitation 5 μg of pBabe (empty vector) or 5 μg of Flag-PRMT5 (generous gift from Dr. Robert Baiocchi’s laboratory, Ohio State University) was co-tranfected with 5 μg of S-p30-HA or S-p28-HA. The cells were harvested and lysed in 1x passive lysis buffer after 24 hr (Promega, Madison, WI). 293T cells treated with siRNA or lentiviral particles expressing scrambled shRNA or PRMT5 shRNA were transfected with the HTLV-1 molecular clone, Ach (1.8 μg/well) and HTLV-2 molecular clone, Ph6neo (1.8 μg/well) of a six well plate using SuperFect (Qiagen, Valencia, CA). Oligofectamine (Invitrogen, Grand Island, NY) was used to transfect 293T cells with 100 nM of control siRNA or smart pool siRNA against REGγ and NIP30 (Dharmacon, Lafayette, CO). Stable knockdowns were produced by transducing 293T cells with lentiviral particles expresssing scramble or PRMT5 shRNAs (generous gift from Dr. Robert Baiocchi, Ohio State University) and selected for puromycin resistance.

### HTLV-1 and HTLV-2 gene expression

Molecular proviral clones of HTLV-1 and HTLV-2 were transfected 24 hrs after siRNA treatment and incubated for 24 hrs. Supernatant was collected for measure of p19 Gag by ELISA (Zeptometrix, Buffalo, NY) and cells were lysated and immunoblotted for p24 production.

### S-tag affinity purification and immunoprecipitation

Cell lysates were prepared with 1X passive lysis buffer (Promega, Madison, WI) in the presence of protease inhibitor mixture (Roche Applied Science, Indianapolis, IN). S-tag purification was performed by rocking cell lysates with S-beads (Novagen, Madison, WI) overnight at 4°C. The S-beads were washed once with high salt (1 M NaCl) containing radioimmunoprecipitation buffer (150 mM NaCl, 0.01 M Sodium pyrophosphate, 10 mM EDTA, 10 mM sodium fluoride, 50 mM Tris, 0.1% SDS, 12.8 mM deoxycholic acid, 10% glycerol and 1% Nonidet P-40 (pH 8.0)), three times with radioimmunoprecipitation buffer and once with PBS. The beads were suspended in ddH_2_O and subjected to shotgun proteomics. For subsequent immunoblotting the proteins were eluted by boiling the beads in SDS loading buffer. Immunoprecipitation was performed by rocking cell lysates overnight with specific antibody at 4°C and then with Sepharose protein A beads for 2 hrs (GE Sweden). Sepharose protein A beads were washed twice with high salt (1 M NaCl) containing radioimmunoprecipitation assay buffer and thrice with low salt (150 nM NaCl) radioimmunoprecipitation assay buffer and once with PBS. For western blotting proteins were eluted by boiling the beads in SDS loading buffer.

### Shotgun proteomics

Capillary-liquid chromatography-tandem mass spectrometry (Capillary-LC/MS/MS) for global protein identification was performed on a Thermo Finnigan LTQ Orbitrap mass spectrometer equipped with a microspray source (Michrom Bioresources Inc, Auburn, CA) operated in positive ion mode. Samples were separated on a capillary column (0.2X150 mm Magic C18AQ 3 μ 200A, Michrom Bioresources Inc, Auburn, CA) using an UltiMate^TM^ 3000 HPLC system from LC-Packings A Dionex Co. (Sunnyvale, CA). The scan sequence of the mass spectrometer was based on the data dependant TopTen^TM^ method. The resolution of a full scan was set at 30000 to achieve a high mass accuracy MS determination.

The RAW data files collected on the mass spectrometer were converted to mzXML and MGF files by use of MassMatrix data conversion tools (version 1.3, http://www.massmatrix.net/download). The resulting MGF files were searched using Mascot Daemon by Matrix Science version 2.2.2 (Boston, MA) and the database searched against the full SwissProt database version 57.5 (471472 sequences; 167326533 residues) or NCBI database version 20091013 (9873339 sequences; 3367482728 residues). Considered modifications (variable) were methionine oxidation and the presence of carbamidomethyl cysteine. Three missed cleavages for the enzyme were permitted with a peptide tolerance of 1.2 Da, and the MS/MS ion tolerance was 0.8 Da. Mock transfected (empty pTriEx4-Neo) cell lysates treated similarly served as negative control. Search results were compiled and visualized using the Scaffold 3 sofware. Unweighted spectrum count and percent coverage provided semi-quantitative data analyses. Protein identifications were assigned using PeptideProphet. Proteins with 80% confidence were accepted with a minimum of one peptide displaying 95% threshold confidence level.

### Immunoblotting and antibodies

To confirm the MS/MS data of selected p28- and p30-interacting proteins we performed immunoblotting analysis. Cell lysate-derived proteins and proteins from S-Tag purification or immunoprecipitation assays were resolved by 4–20% gradient SDS-PAGE and transferred to nitrocellulose membranes prior to immunoblotting using the following primary and secondary antibodies: mouse anti-HA monoclonal antibodies (1:1000) (Covance Research Products, Princeton, NJ); rabbit anti-hnRNPH1 monoclonal (1:1000), mouse anti-hnRNPH1 (1:1000), mouse anti-HTLV p24 (1:1000) (Abcam, Cambridge, MA); Rabbit anti-NIP30 monoclonal (1:1000), rabbit anti-REGγ (1:1000) (Proteintech Group, Chicago, IL); mouse-anti β-actin (1:2000) (Sigma-Aldrich, St. Louis, MO); rat anti-Flag (1:1000) horse anti-mouse (1:2000), and goat anti-rabbit antibodies (1:2000) anti-rat (1:2000) (Cell Signaling, Danvers, MA).

## Abbreviations

NIP30, NEFA interacting protein 30; PRMT5, Protein Arginine methlytransferase 5; hnRNP H1, Heterogeneous nuclear ribonucleoprotein H1; HTLV-1 and -2, Human T-cell lymphotropic virus type 1 and 2; ATL, Adult T cell leukemia; HAM/TSP, HTLV-1 associated myelopathy/tropical spastic paraparesis.

## Misc

Rami Doueiri and Rajaneesh Anupam contributed equally to this work.

## Competing interests

Authors have no competing interests involved in data collection, materials and methods or conclusions.

## Authors’ contributions

RD and RA contributed equally to designing and collecting the data presented in the paper. KBG headed the shot-proteomics data collection. MK helped with mass spectrometry-based proteomic data analysis and finalizing the manuscript. MDL and PLG conceived, coordinated and helped in writing the manuscript. All authors read and approved the final manuscript.

## Supplementary Material

Additional file 1 **Figure S1. Effects of REGγ and NIP30 knockdown on HTLV-1 gene expression.** A) Two independent experiments (labeled 1 and 2) of control and REGγ siRNA treated 293T cells were transfected with Ach (HTLV-1 molecular clone). The knockdown of REGγ was confirmed by immunoblotting with anti-REGγ. The expression of HTLV p24 was monitored by immunoblotting. B) Negative siRNA and NIP30 siRNA treated 293 T cells were transfected with HTLV-1 molecular clone (Ach). The levels of NIP30 were tested by immunoblotting with anti-NIP30 antibodies. The expression of HTLV-1 p24 was monitored by anti-HTLV-1 p24 antibody. Equal loading of samples was confirmed by anti-actin antibodies. (JPEG 51 kb) Click here for file

## References

[B1] Manns A Blattner WA The epidemiology of the human T-cell lymphotrophic virus type I and type II: etiologic role in human disease Transfusion 1991 31 67 75 1986467 10.1046/j.1537-2995.1991.31191096189.x

[B2] Poiesz BJ Ruscetti FW Gazdar AF Bunn PA Minna JD Gallo RC Detection and isolation of type C retrovirus particles from fresh and cultured lymphocytes of a patient with cutaneous T-cell lymphoma Proc Natl Acad Sci USA 1980 77 7415 7419 6261256 10.1073/pnas.77.12.7415 PMC350514

[B3] Yoshida M Miyoshi I Hinuma Y Isolation and characterization of retrovirus from cell lines of human adult T-cell leukemia and its implication in the disease Proc Natl Acad Sci USA 1982 79 2031 2035 6979048 10.1073/pnas.79.6.2031 PMC346116

[B4] Koyanagi Y Itoyama Y Nakamura N Takamatsu K Kira J Iwamasa T Goto I Yamamoto N In vivo infection of human T-cell leukemia virus type I in non-T cells Virology 1993 196 25 33 8356797 10.1006/viro.1993.1451

[B5] Jones KS Petrow-Sadowski C Huang YK Bertolette DC Ruscetti FW Cell-free HTLV-1 infects dendritic cells leading to transmission and transformation of CD4(+) T cells Nat Med 2008 14 429 436 18376405 10.1038/nm1745

[B6] Yasunaga J Sakai T Nosaka K Etoh K Tamiya S Koga S Mita S Uchino M Mitsuya H Matsuoka M Impaired production of naive T lymphocytes in human T-cell leukemia virus type I-infected individuals: its implications in the immunodeficient state Blood 2001 97 3177 3183 11342446 10.1182/blood.v97.10.3177

[B7] Takatsuki K Discovery of adult T-cell leukemia Retrovirology 2005 2 16 15743528 10.1186/1742-4690-2-16 PMC555581

[B8] Bangham CR Osame M Cellular immune response to HTLV-1 Oncogene 2005 24 6035 6046 16155610 10.1038/sj.onc.1208970

[B9] Murphy EL Fridey J Smith JW Engstrom J Sacher RA Miller K Gibble J Stevens J Thomson R Hansma D HTLV-associated myelopathy in a cohort of HTLV-I and HTLV-II-infected blood donors. The REDS investigators Neurology 1997 48 315 320 9040713 10.1212/wnl.48.2.315

[B10] Jones KS Fugo K Petrow-Sadowski C Huang Y Bertolette DC Lisinski I Cushman SW Jacobson S Ruscetti FW Human T-cell leukemia virus type 1 (HTLV-1) and HTLV-2 use different receptor complexes to enter T cells J Virol 2006 80 8291 8302 16912281 10.1128/JVI.00389-06 PMC1563841

[B11] Iacampo S Cochrane A Human immunodeficiency virus type 1 Rev function requires continued synthesis of its target mRNA J Virol 1996 70 8332 8339 8970952 10.1128/jvi.70.12.8332-8339.1996 PMC190920

[B12] Ijichi S Ramundo MB Takahashi H Hall WW In vivo cellular tropism of human T-cell leukemia virus type II (HTLV-II) J Exp Med 1992 176 293 296 1351922 10.1084/jem.176.1.293 PMC2119301

[B13] Xie L Green PL Envelope is a major viral determinant of the distinct in vitro cellular transformation tropism of human T-cell leukemia virus type 1 (HTLV-1) and HTLV-2 J Virol 2005 79 14536 14545 16282453 10.1128/JVI.79.23.14536-14545.2005 PMC1287554

[B14] Collins ND Newbound GC Ratner L Lairmore MD In vitro CD4 + lymphocyte transformation and infection in a rabbit model with a molecular clone of human T-cell lymphotropic virus type 1 J Virol 1996 70 7241 7246 8794375 10.1128/jvi.70.10.7241-7246.1996 PMC190781

[B15] Wang T-G Ye J Lairmore M Green PL In vitro cellular tropism of human T-cell leukemia virus type 2 AIDS Res Hum Retroviruses 2000 16 1661 1668 11080807 10.1089/08892220050193119

[B16] Grassmann R Aboud M Jeang KT Molecular mechanisms of cellular transformation by HTLV-1 Tax Oncogene 2005 24 5976 5985 16155604 10.1038/sj.onc.1208978

[B17] Ross TM Pettiford SM Green PL The tax gene of human T-cell leukemia virus type 2 is essential for transformation of human T lymphocytes J Virol 1996 70 5194 5202 8764028 10.1128/jvi.70.8.5194-5202.1996 PMC190475

[B18] Feuer G Green PL Comparative biology of human T-cell lynphotropic virus type 1 (HTLV-1) and HTLV-2 Oncogene 2005 24 5996 6004 16155606 10.1038/sj.onc.1208971 PMC2659530

[B19] Gaudray G Gachon F Basbous J Biard-Piechaczyk M Devaux C Mesnard J The complementary strand of the human T-cell leukemia virus type 1 RNA genome encodes a bZIP transcription factor that down-regulates viral transcription J Virol 2002 76 12813 12822 12438606 10.1128/JVI.76.24.12813-12822.2002 PMC136662

[B20] Halin M Douceron E Clerc I Journo C Ko NL Landry S Murphy EL Gessain A Lemasson I Mesnard JM Human T-cell leukemia virus type 2 produces a spliced antisense transcript encoding a protein that lacks a classic bZIP domain but still inhibits Tax2-mediated transcription Blood 2009 114 2427 2438 19602711 10.1182/blood-2008-09-179879 PMC2746472

[B21] Matsuoka M Green PL The HBZ gene, a key player in HTLV-1 pathogenesis Retrovirology 2009 6 71 19650892 10.1186/1742-4690-6-71 PMC2731725

[B22] Nicot C Dundr JM Johnson JR Fullen JR Alonzo N Fukumoto R Princler GL Derse D Misteli T Franchini G HTLV-1-encoded p30II is a post-transcriptional negative regulator of viral replication Nat Med 2004 10 197 201 14730358 10.1038/nm984

[B23] Zhang W Nisbet JW Albrecht B Ding W Kashanchi F Bartoe JT Lairmore MD Human T-lymphotropic virus type 1 p30II regulates gene transcription by binding CREB binding protein/p300 J Virol 2001 75 9885 9895 11559821 10.1128/JVI.75.20.9885-9895.2001 PMC114560

[B24] Younis I Boris-Lawrie K Green PL Human T-cell leukemia virus ORF II p28 encodes a post-transcriptional repressor that is recruited at the level of transcription J Virol 2006 80 181 191 16352542 10.1128/JVI.80.1.181-191.2006 PMC1317543

[B25] Silverman LR Phipps AJ Montgomery A Ratner L Lairmore MD Human T-cell lymphotropic virus type 1 open reading frame II-encoded p30II is required for in vivo replication: evidence of in vivo reversion J Virol 2004 78 3837 3845 15047799 10.1128/JVI.78.8.3837-3845.2004 PMC374265

[B26] Valeri VW Hryniewicz A Andresen V Jones K Fenizia C Bialuk I Chung HK Fukumoto R Washington Parks R Ferrari MG Requirement of the human T-cell leukemia virus p12 and p30 genes for infectivity of human dendritic cells and macaques but not rabbits Blood 2010 10.1182/blood-2010-05-284141 PMC2981536 20647569

[B27] Yamamoto B Li M Kesic M Younis I Lairmore MD Green PL Human T-cell leukemia virus type 2 post-transcriptional control protein p28 is required for viral infectivity and persistence in vivo Retrovirology 2008 5 38 18474092 10.1186/1742-4690-5-38 PMC2405800

[B28] Robek M Wong F Ratner L Human T-cell leukemia virus type 1 pX-I and pX-II open reading frames are dispensable for the immortalization of primary lymphocytes J Virol 1998 72 4458 4462 9557741 10.1128/jvi.72.5.4458-4462.1998 PMC109681

[B29] Koralnik IJ Fullen J Franchini G The p12I, p13II, and p30II proteins encoded by human T-cell leukemia/lymphotropic virus type I open reading frames I and II are localized in three different cellular compartments J Virol 1993 67 2360 2366 8445734 10.1128/jvi.67.4.2360-2366.1993 PMC240398

[B30] Younis I Khair L Dundr M Lairmore MD Franchini G Green PL Repression of human T-cell leukemia virus type 1 and 2 replication by a viral mRNA-encoded posttranscriptional regulator J Virol 2004 78 11077 11083 15452228 10.1128/JVI.78.20.11077-11083.2004 PMC521841

[B31] Ghorbel S Sinha-Datta U Dundr M Brown M Franchini G Nicot C Human T-cell leukemia virus type I p30 nuclear/nucleolar retention is mediated through interactions with RNA and a constituent of the 60 S ribosomal subunit J Biol Chem 2006 281 37150 37158 17008317 10.1074/jbc.M603981200

[B32] Sinha-Datta U Datta A Ghorbel S Dodon MD Nicot C Human T-cell Lymphotrophic Virus Type I Rex and p30 Interactions Govern the Switch between Virus Latency and Replication J Biol Chem 2007 282 14608 14615 17360706 10.1074/jbc.M611219200

[B33] Michael B Nair AM Hiraragi H Shen L Feuer G Boris-Lawrie K Lairmore MD Human T lymphotropic virus type 1 p30II alters cellular gene expression to selectively enhance signaling pathways that activate T lymphocytes Retrovirology 2004 1 39 15560845 10.1186/1742-4690-1-39 PMC538277

[B34] Taylor JM Ghorbel S Nicot C Genome wide analysis of human genes transcriptionally and post-transcriptionally regulated by the HTLV-I protein p30 BMC Genomics 2009 10 311 19602286 10.1186/1471-2164-10-311 PMC2723137

[B35] Datta A Silverman L Phipps AJ Hiraragi H Ratner L Lairmore MD Human T-lymphotropic virus type-1 p30 alters cell cycle G2 regulation of T lymphocytes to enhance cell survival Retrovirology 2007 4 49 17634129 10.1186/1742-4690-4-49 PMC1937004

[B36] Baydoun HH Pancewicz J Bai X Nicot C HTLV-I p30 inhibits multiple S phase entry checkpoints, decreases cyclin E-CDK2 interactions and delays cell cycle progression Mol Canc 2010 9 302 10.1186/1476-4598-9-302 PMC3000403 21092281

[B37] Anupam R Datta A Kesic M Green-Church K Shkriabai N Kvaratskhelia M Lairmore MD Human T-lymphotropic virus type 1 p30 interacts with REGgamma and modulates ATM (ataxia telangiectasia mutated) to promote cell survival J Biol Chem 2011 286 7661 7668 21216954 10.1074/jbc.M110.176354 PMC3045020

[B38] Awasthi S Sharma A Wong K Zhang J Matlock EF Rogers L Motloch P Takemoto S Taguchi H Cole MD A Human T-Cell Lymphotropic Virus Type 1 Enhancer of Myc Transforming Potential Stabilizes Myc-TIP60 Transcriptional Interactions Mol Cell Biol 2005 25 6178 6198 15988028 10.1128/MCB.25.14.6178-6198.2005 PMC1168837

[B39] Baydoun HH Pancewicz J Nicot C Human T-lymphotropic type 1 virus p30 inhibits homologous recombination and favors unfaithful DNA repair Blood 2011 117 5897 5906 21427292 10.1182/blood-2010-08-304600 PMC3112037

[B40] Barrios CS Abuerreish M Lairmore MD Castillo L Giam CZ Beilke MA Recombinant human T-cell leukemia virus types 1 and 2 Tax proteins induce high levels of CC-chemokines and downregulate CCR5 in human peripheral blood mononuclear cells Viral Immunol 2011 24 429 439 22111594 10.1089/vim.2011.0037 PMC3234705

[B41] Bertazzoni U Turci M Avesani F Di Gennaro G Bidoia C Romanelli MG Intracellular localization and cellular factors interaction of HTLV-1 and HTLV-2 Tax proteins: similarities and functional differences Viruses 2011 3 541 560 21994745 10.3390/v3050541 PMC3185761

[B42] Higuchi M Fujii M Distinct functions of HTLV-1 Tax1 from HTLV-2 Tax2 contribute key roles to viral pathogenesis Retrovirology 2009 6 117 20017952 10.1186/1742-4690-6-117 PMC2806368

[B43] Raines RT McCormick M Van Oosbree TR Mierendorf RC The S. Tag fusion system for protein purification Methods Enzymol 2000 326 362 376 11036653 10.1016/s0076-6879(00)26065-5

[B44] Nishioka K Reinberg D Methods and tips for the purification of human histone methyltransferases Methods 2003 31 49 58 12893173 10.1016/s1046-2023(03)00087-2

[B45] Simonis N Rual JF Lemmens I Boxus M Hirozane-Kishikawa T Gatot JS Dricot A Hao T Vertommen D Legros S Host-pathogen interactome mapping for HTLV-1 and −2 retroviruses Retrovirology 2012 9 26 22458338 10.1186/1742-4690-9-26 PMC3351729

[B46] Lairmore MD Anupam R Bowden N Haines R Haynes RA Ratner L Green PL Molecular determinants of human T-lymphotropic virus type 1 transmission and spread Viruses 2011 3 1131 1165 21994774 10.3390/v3071131 PMC3185783

[B47] Michael B Nair AM Datta A Hiraragi H Ratner L Lairmore MD Histone acetyltransferase (HAT) activity of p300 modulates human T lymphotropic virus type 1 p30II-mediated repression of LTR transcriptional activity Virology 2006 354 225 239 16890266 10.1016/j.virol.2006.07.002 PMC3044896

[B48] Rechsteiner M Hill CP Mobilizing the proteolytic machine: cell biological roles of proteasome activators and inhibitors Trends Cell Biol 2005 15 27 33 15653075 10.1016/j.tcb.2004.11.003

[B49] Li X Amazit L Long W Lonard DM Monaco JJ O'Malley BW Ubiquitin- and ATP-independent proteolytic turnover of p21 by the REGgamma-proteasome pathway Mol Cell 2007 26 831 842 17588518 10.1016/j.molcel.2007.05.028

[B50] Chen X Barton LF Chi Y Clurman BE Roberts JM Ubiquitin-independent degradation of cell-cycle inhibitors by the REGgamma proteasome Mol Cell 2007 26 843 852 17588519 10.1016/j.molcel.2007.05.022 PMC2031223

[B51] Zhang Z Zhang R Proteasome activator PA28 gamma regulates p53 by enhancing its MDM2-mediated degradation EMBO J 2008 27 852 864 18309296 10.1038/emboj.2008.25 PMC2265109

[B52] Li X Lonard DM Jung SY Malovannaya A Feng Q Qin J Tsai SY Tsai MJ O'Malley BW The SRC-3/AIB1 coactivator is degraded in a ubiquitin- and ATP-independent manner by the REGgamma proteasome Cell 2006 124 381 392 16439211 10.1016/j.cell.2005.11.037

[B53] Barnikol-Watanabe S Gross NA Gotz H Henkel T Karabinos A Kratzin H Barnikol HU Hilschmann N Human protein NEFA, a novel DNA binding/EF-hand/leucine zipper protein. Molecular cloning and sequence analysis of the cDNA, isolation and characterization of the protein Biol Chem Hoppe Seyler 1994 375 497 512 7811391 10.1515/bchm3.1994.375.8.497

[B54] Nesselhut J Jurgan U Onken E Gotz H Barnikol HU Hirschfeld G Barnikol-Watanabe S Hilschmann N Golgi retention of human protein NEFA is mediated by its N-terminal Leu/Ile-rich region FEBS Lett 2001 509 469 475 11749975 10.1016/s0014-5793(01)03187-8

[B55] Simpson JC Wellenreuther R Poustka A Pepperkok R Wiemann S Systematic subcellular localization of novel proteins identified by large-scale cDNA sequencing EMBO Rep 2000 1 287 292 11256614 10.1093/embo-reports/kvd058 PMC1083732

[B56] Feske S Calcium signalling in lymphocyte activation and disease Nat Rev Immunol 2007 7 690 702 17703229 10.1038/nri2152

[B57] Paul S Dansithong W Kim D Rossi J Webster NJ Comai L Reddy S Interaction of muscleblind, CUG-BP1 and hnRNP H proteins in DM1-associated aberrant IR splicing EMBO J 2006 25 4271 4283 16946708 10.1038/sj.emboj.7601296 PMC1570429

[B58] Zhang Y Lindblom T Chang A Sudol M Sluder AE Golemis EA Evidence that dim1 associates with proteins involved in pre-mRNA splicing, and delineation of residues essential for dim1 interactions with hnRNP F and Npw38/PQBP-1 Gene 2000 257 33 43 11054566 10.1016/s0378-1119(00)00372-3

[B59] Ostrowski J Van Seuningen I Seger R Rauch CT Sleath PR McMullen BA Bomsztyk K Purification, cloning, and expression of a murine phosphoprotein that binds the kappa B motif in vitro identifies it as the homolog of the human heterogeneous nuclear ribonucleoprotein K protein. Description of a novel DNA-dependent phosphorylation process J Biol Chem 1994 269 17626 17634 8021272

[B60] Di Lorenzo A Bedford MT Histone arginine methylation FEBS Lett 2011 585 2024 2031 21074527 10.1016/j.febslet.2010.11.010 PMC3409563

[B61] Wang L Pal S Sif S Protein arginine methyltransferase 5 suppresses the transcription of the RB family of tumor suppressors in leukemia and lymphoma cells Mol Cell Biol 2008 28 6262 6277 18694959 10.1128/MCB.00923-08 PMC2577430

[B62] Hou Z Peng H Ayyanathan K Yan KP Langer EM Longmore GD Rauscher FJ The LIM protein AJUBA recruits protein arginine methyltransferase 5 to mediate SNAIL-dependent transcriptional repression Mol Cell Biol 2008 28 3198 3207 18347060 10.1128/MCB.01435-07 PMC2423142

[B63] Tabata T Kokura K Ten Dijke P Ishii S Ski co-repressor complexes maintain the basal repressed state of the TGF-beta target gene, SMAD7, via HDAC3 and PRMT5 Genes Cells 2009 14 17 28 19032343 10.1111/j.1365-2443.2008.01246.x

[B64] Cesaro E De Cegli R Medugno L Florio F Grosso M Lupo A Izzo P Costanzo P The Kruppel-like zinc finger protein ZNF224 recruits the arginine methyltransferase PRMT5 on the transcriptional repressor complex of the aldolase A gene J Biol Chem 2009 284 32321 32330 19741270 10.1074/jbc.M109.043349 PMC2781646

[B65] Pal S Vishwanath SN Erdjument-Bromage H Tempst P Sif S Human SWI/SNF-associated PRMT5 methylates histone H3 arginine 8 and negatively regulates expression of ST7 and NM23 tumor suppressor genes Mol Cell Biol 2004 24 9630 9645 15485929 10.1128/MCB.24.21.9630-9645.2004 PMC522266

[B66] Lacroix M El Messaoudi S Rodier G Le Cam A Sardet C Fabbrizio E The histone-binding protein COPR5 is required for nuclear functions of the protein arginine methyltransferase PRMT5 EMBO Rep 2008 9 452 458 18404153 10.1038/embor.2008.45 PMC2373370

[B67] Scoumanne A Zhang J Chen X PRMT5 is required for cell-cycle progression and p53 tumor suppressor function Nucleic Acids Res 2009 37 4965 4976 19528079 10.1093/nar/gkp516 PMC2731901

[B68] Jansson M Durant ST Cho EC Sheahan S Edelmann M Kessler B La Thangue NB Arginine methylation regulates the p53 response Nat Cell Biol 2008 10 1431 1439 19011621 10.1038/ncb1802

[B69] Martin G Ostareck-Lederer A Chari A Neuenkirchen N Dettwiler S Blank D Ruegsegger U Fischer U Keller W Arginine methylation in subunits of mammalian pre-mRNA cleavage factor I RNA 2010 16 1646 1659 20562214 10.1261/rna.2164210 PMC2905762

[B70] Neuenkirchen N Chari A Fischer U Deciphering the assembly pathway of Sm-class U snRNPs FEBS Lett 2008 582 1997 2003 18348870 10.1016/j.febslet.2008.03.009

