# Supplementary figures and images for: Comparative host protein interactions with HTLV-1 p30 and HTLV-2 p28: insights into difference in pathobiology of human retroviruses
Source: Retrovirology. 2012 Aug 9;9:64. doi: 10.1186/1742-4690-9-64 (PMC3464894; doi:10.1186/1742-4690-9-64)

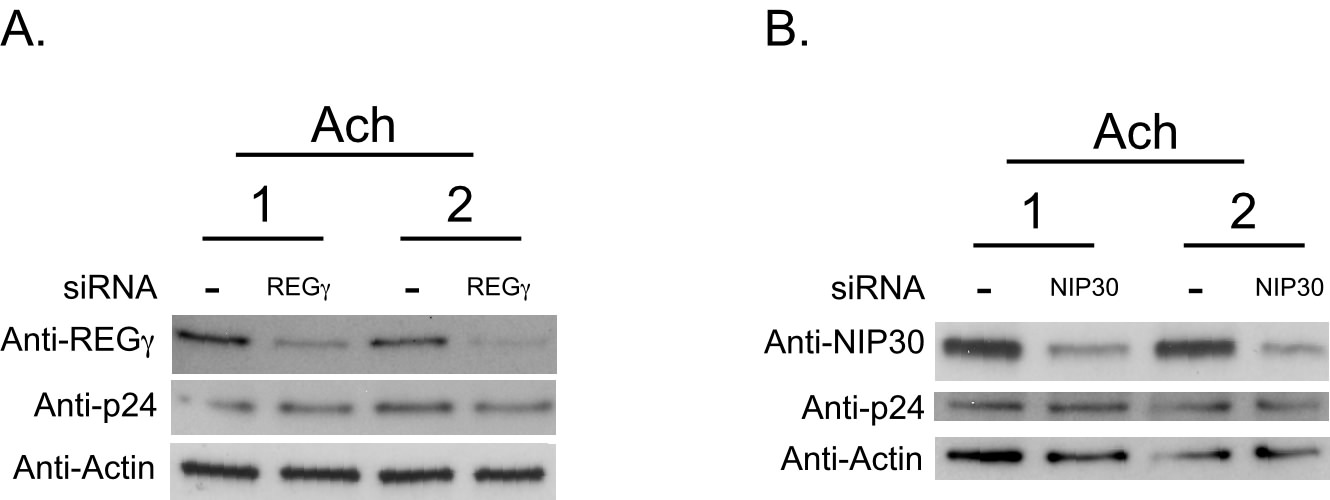

Supplement: Additional file 1 — Figure S1. Effects of REGγ and NIP30 knockdown on HTLV-1 gene expression. A) Two independent experiments (labeled 1 and 2) of control and REGγ siRNA treated 293T cells were transfected with Ach (HTLV-1 molecular clone). The knockdown of REGγ was confirmed by immunoblotting with anti-REGγ. The expression of HTLV p24 was monitored by immunoblotting. B) Negative siRNA and NIP30 siRNA treated 293 T cells were transfected with HTLV-1 molecular clone (Ach). The levels of NIP30 were tested by immunoblotting with anti-NIP30 antibodies. The expression of HTLV-1 p24 was monitored by anti-HTLV-1 p24 antibody. Equal loading of samples was confirmed by anti-actin antibodies. (JPEG 51 kb) [file 1742-4690-9-64-S1.jpeg]
